# AptBCis1, An Aptamer–Cisplatin Conjugate, Is Effective in Lung Cancer Leptomeningeal Carcinomatosis

**DOI:** 10.1021/acsnano.4c04680

**Published:** 2024-10-03

**Authors:** Bo-Tsang Huang, Wei-Yun Lai, Chen-Lin Yeh, Yi-Ting Tseng, Konan Peck, Pan-Chyr Yang, Emily Pei-Ying Lin

**Affiliations:** † Division of Thoracic Medicine, Department of Internal Medicine, School of Medicine, College of Medicine, Taipei Medical University, Taipei 110, Taiwan; ‡ Division of Thoracic Medicine, Department of Internal Medicine, Taipei Medical University Hospital, Taipei 110, Taiwan; § Department of Medical Research, Taipei Medical University Hospital, Taipei 110, Taiwan; ∥ Institute of Biomedical Sciences, Academia Sinica, Taipei 115, Taiwan; ¶ Division of Chest Medicine, Department of Internal Medicine, National Taiwan University Hospital and College of Medicine, Taipei 100, Taiwan

**Keywords:** lung cancer, leptomeningeal carcinomatosis, blood−brain barrier, aptamer, cisplatin, *in vivo* SELEX

## Abstract

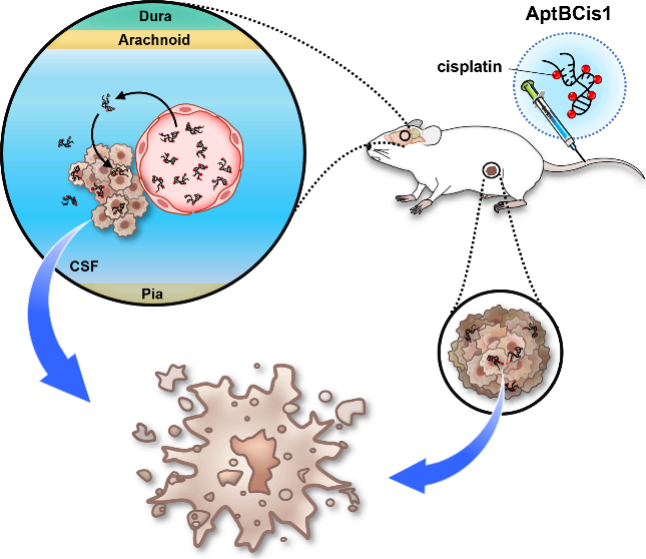

Treatment of lung cancer leptomeningeal carcinomatosis (LM) remains challenging partly due to the biological nature of the blood–brain barrier (BBB). Cisplatin has limited effects on LM, and it is notorious for neurotoxicity. Aptamers are small oligonucleotides considered as antibody surrogates. Here we report a DNA therapeutics, AptBCis1. AptBCis1 is a cisplatin-conjugated, BBB-penetrating, and cancer-targeting DNA aptamer. Its backbone, AptB1, was identified via *in vivo* SELEX using lung cancer LM orthotopic mouse models. The AptB1 binds to EAAT2, Nucleolin, and YB-1 proteins. Treatment with AptBCis1 1 mg/kg (equivalent to cisplatin 0.35 mg/kg) showed superior tumor suppressive effects compared to cisplatin 2 mg/kg in mice with lung cancer LM diseases. The cerebrospinal fluid platinum concentration in the AptBCis1 group was 10% of that in the cisplatin group. The data suggested the translational potential of AptBCis1 in lung cancer with LM and in cancers in which platinum-based chemotherapy remains as the standard of care.

Leptomeningeal carcinomatosis (LM) was reported in 3–10% of patients with advanced-stage lung cancer and was found in 20% of patients with solid tumors at autopsy.^1,2^ However, options for treatment are limited, partly due to the existence of the blood–brain barrier (BBB) that restrains effective drug delivery into the central nervous system (CNS).^1−3^

The BBB dynamically regulates brain homeostasis. It is composed of brain capillary endothelial cells, pericytes, astrocytic foot processes, and nerve endings terminating on the capillary surface. Tight and adherent junctions between the adjacent endothelial cells prohibit paracellular transport of hydrophilic compounds across the BBB, and transcellular transport by passive diffusion is available only to lipophilic or water-soluble molecules under 500 Da. Moreover, the active efflux transporters on the BBB restrict the entry of chemotherapeutic agents into the CNS.^3^

Cisplatin is a platinum compound that constitutes the backbone for lung cancer chemotherapy regimens.^4,5^ Cisplatin exerts anticancer activity through multiple mechanisms, and the induction of DNA damage response is the most prominent mode of action.^6^ However, the drug is notorious for neurotoxicity, a side effect that limits its maximum tolerated cumulative dose.^7^ Moreover, although the systemic response rate of cisplatin achieves up to 21%, it has minimal effects on the LM diseases.^1,2,8^ This could be due to its inadequate intratumor concentrations. Therefore, precision and effective delivery of the drug are important, especially for cancers with LM.

Aptamers are small oligonucleotides folded into 3D structures. Aptamers can bind to proteins with high affinity and are considered as antibody surrogates.^9^ Compared with monoclonal antibodies, aptamers are small and may possess advantages for targeted delivery to certain tissue compartments, for example, the CNS.^9,10^ Recent studies showed the potentiality of aptamers in neuro-oncology and neurodegenerative disorders.^11,12^ Nevertheless, no aptamer therapeutics for LM diseases have yet been reported.

In the current study, we reported a DNA therapeutics, AptBCis1. AptBCis1 is a cisplatin-conjugated, BBB-penetrating, and cancer-targeting DNA aptamer. An *in vivo* SELEX platform, the lung cancer LM orthotopic mouse model, was established for the identification of its DNA backbone, AptB1. We also explored potential mechanisms related to the promising tumor suppressive effects of AptBCis1 observed in lung cancer LM orthotopic and subcutaneous xenograft models.

## Results and Discussion

### Establishment of a Leptomeningeal Carcinomatosis (LM) Mouse Model for *In Vivo* SELEX

**Figure 1:**
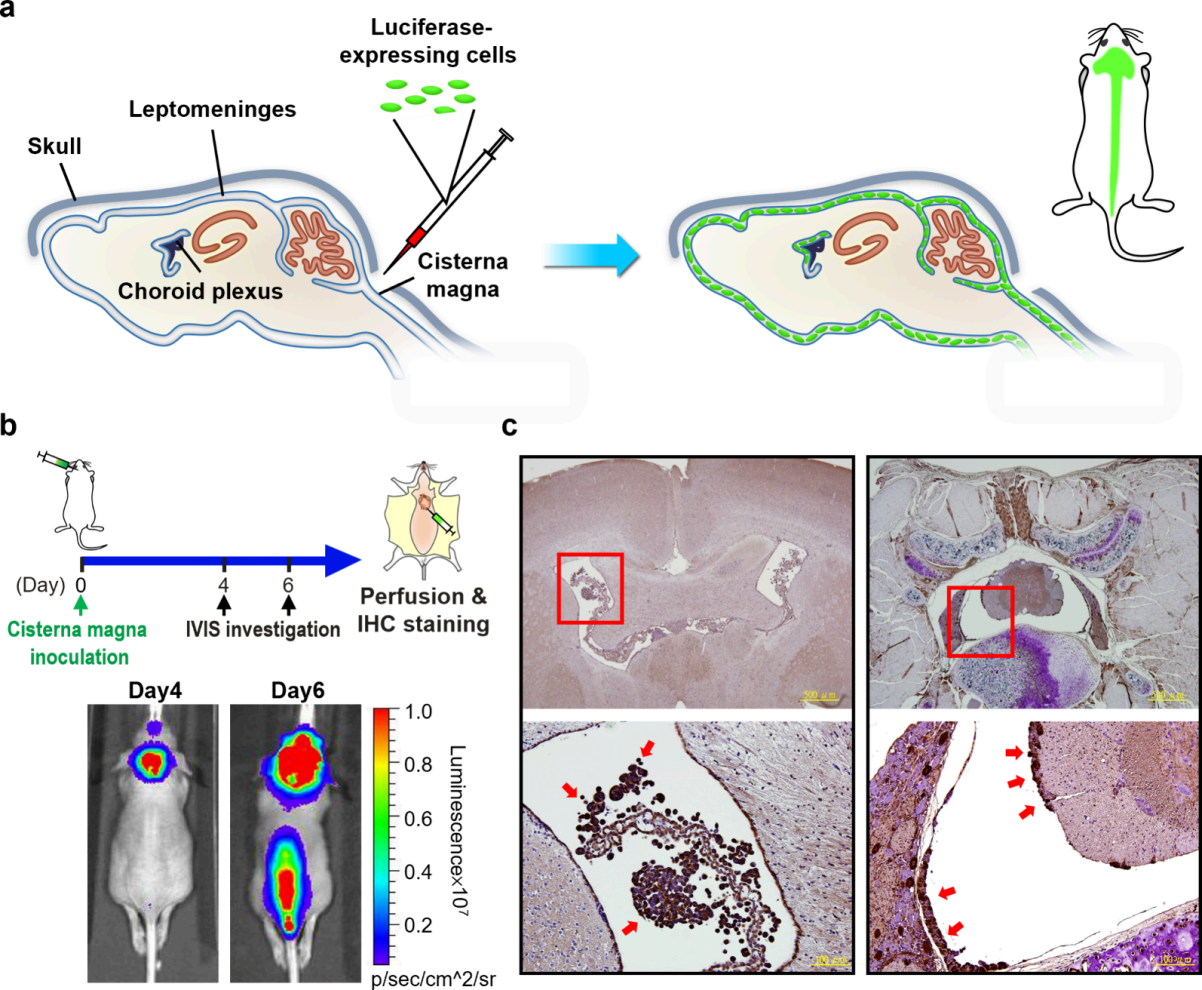
Establishment of a leptomeningeal carcinomatosis (LM) mouse model. (a) Scheme illustrating the direct inoculation of luciferase-expressing cells through cisterna magna to establish a LM orthotopic mouse model. (b) Monitoring of the mouse by IVIS. Prominent BLI signals over the brain and the spinal cord were observed at Day 6 post tumor cell inoculation, and the mouse was sacrificed for IHC confirmation. (c) Tumor cells (red arrows) observed in the ventricular space of the brain (left panel) and the spinal cord (right panel) in IHC studies.

To identify BBB-penetrating and cancer-targeting aptamers, we established a lung cancer LM orthotopic mouse model for *in vivo* SELEX. In brief, luciferase-expressing CL1-5 lung cancer stable cells were inoculated directly into the mouse cisterna magna (Figure 1a). Tumor burden was monitored daily with *in vivo* imaging system (IVIS). The mouse was sacrificed on Day 6 post tumor cell inoculation, at which time point strong bioluminescent (BLI) signals emitted from the tumor cells were detected by the IVIS over anatomical locations of the brain and the spine (Figure 1b). The isolated brain and spinal cord were made into paraffin blocks and were examined with H&E and immunohistochemistry (IHC) stains using antiluciferase antibody. As shown in Figure 1c, tumor cells formed tumor islets on the ventricular cavity walls of the brain (left panel) and the spine (right panel). The data indicated the successful establishment of a lung cancer LM orthotopic mouse model, which served as the *in vivo* SELEX platform for BBB-penetrating aptamer identification.

### Identification of AptB1, a BBB-Penetrating and Cancer-Targeting Aptamer, via *In Vivo* SELEX

**Figure 2:**
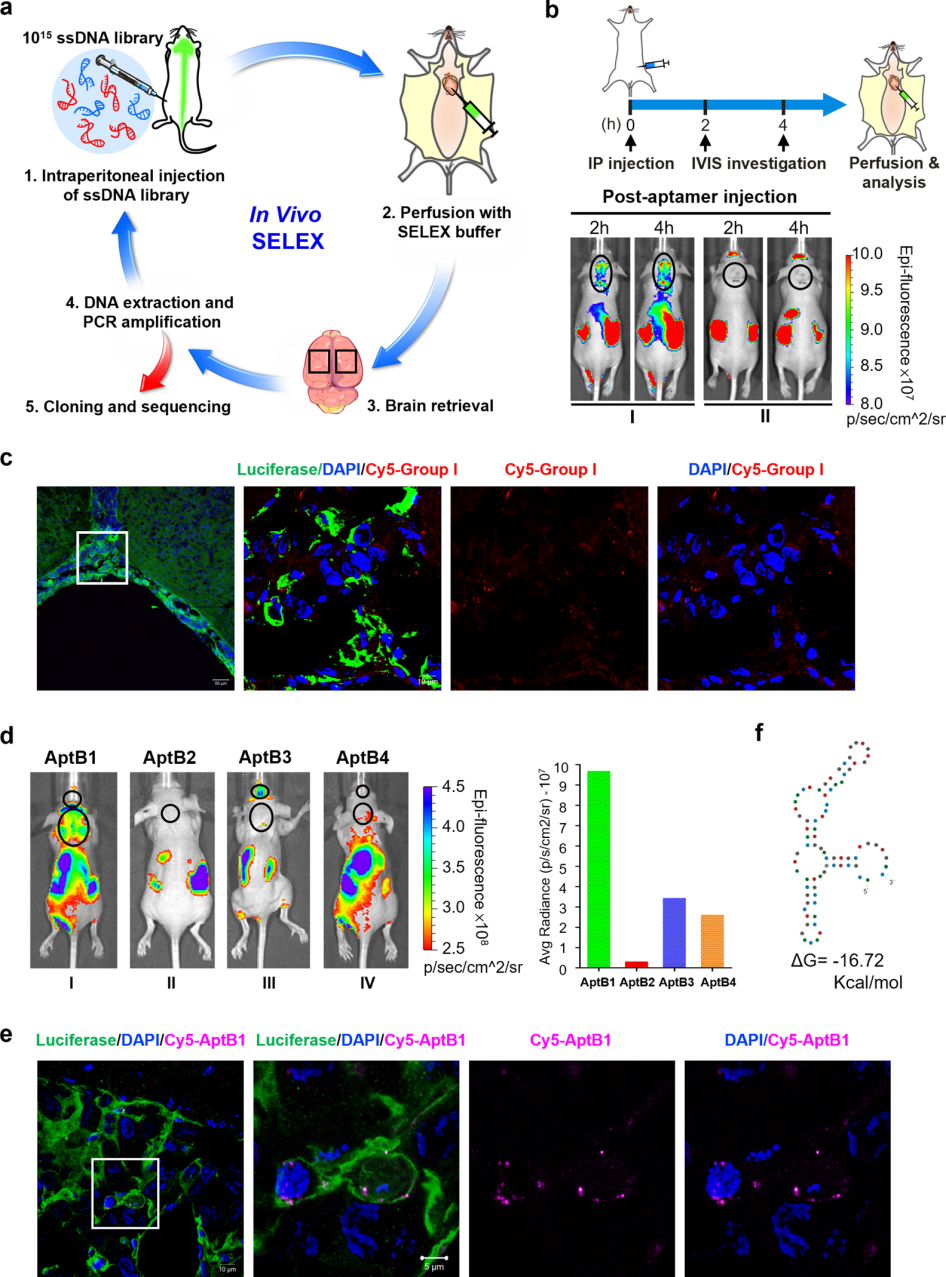
Identification of AptB1, a BBB-penetrating and cancer-targeting aptamer by *in vivo* SELEX. (a) Scheme illustrating the process of *in vivo* SELEX. (b) The aptamers were divided into group I or II based on the QGRS prediction. The group I aptamer contained G-quadruplex structure and the group II did not. The LM mouse I administered with Cy5-labeled group I aptamers showed fluorescent signals over the brain/spine in a crescendo pattern at 2 and 4 h after injection. The lesions are outlined with black circles. (c) Confocal microscopy images revealing group I aptamer signals (red) within the tumor cells (green) on the leptomeninges. (d) Strong Cy5 fluorescent signals emitted from the brain and the spine were detected in the LM mouse administered with AptB1, as shown in the IVIS imaging and the quantified bar chart. The lesions are outlined with black circles. (e) The confocal microscopy images revealed AptB1 signals (pink) within the tumor cell (green) on the leptomeninges. (f) The Mfold prediction of AptB1 secondary structures.

For candidate aptamer identification, an oligonucleotide library, comprised of 10^15^ 80-nucleotide-long ssDNA molecules, was injected intraperitoneally (IP) into the LM mouse. In each SELEX round, the brain was isolated after SELEX buffer perfusion at 6 h post ssDNAs injection, and the brain was isolated for DNA extraction. The extracted DNAs were amplified by PCR with specific primers designed for aptamer amplification. The amplicons were captured by beads and were subjected to single-strand isolation by heating. The ssDNAs were then IP injected again, serving as the library for the subsequent SELEX round. A total of 4 SELEX rounds were carried out, and the enriched oligonucleotide sequences were subjected to Sanger sequencing (Figure 2a). Overall, 9 candidate aptamers (AptB1–9) were identified (Table S1).

The 9 candidate aptamers were amplified by PCR and labeled with the fluorophore Cyanine-5 (Cy5). These aptamers were assigned into two groups based on the QGRS Mapper prediction results: the group I (AptB1–4) sequences contained G-quadruplex structures, while the group II (AptB5–9) sequences did not. The pooled group I or group II aptamers, respectively, were IP injected into the LM mouse I or II, of which prominent BLI signals emitted from the tumor cells were detected over the anatomical location of the brain (Figure S1a). As shown in Figure 2b, strong Cy5 fluorescent signals emitted from the anatomical locations of brain and spine were detected by the IVIS in the LM mouse I, with a crescendo pattern after IP injection, but not in the mouse II. The data suggested the BBB-penetrating ability of the group I aptamers. For confirmation, the LM mouse I was perfused with SELEX buffer and the brain was made into cryosections. As shown in Figure 2c, signals for the group I aptamers (red) were detected in the tumor cells (green) on the leptomeninges. The data further supported the BBB-penetrating ability of the group I aptamers and suggested their cancer-targeting potentiality.

Next, the 4 aptamers within group I, Cy5-AptB1 to Cy5-AptB4, were individually IP injected into the LM mice. The data showed the detection of Cy5 signals over the anatomical locations of the brain and/or spine in mice injected with AptB1 and AptB3, but not AptB2 or AptB4 (Figure 2d and Figure S1b); signals emitted from the tumor cells are shown in the Figure S1c. As the AptB1-injected mouse showed the most promising signal, the mouse brain was made into cryosections for further confirmation. As expected, confocal microscopy images revealed AptB1 signals (pink) within the tumor cells (green) on the leptomeninges, suggesting its BBB-penetrating and cancer-targeting ability (Figure 2e). A structure prediction by Mfold suggested that AptB1 formed 3 stem-loops with a total of 9 GC pairs (Figure 2f).

To confirm the BBB-penetrating ability of AptB1, it was IP administered into tumor-naïve nude mice. As shown in Figure S1d, a crescendo pattern of the Cy5-AptB1 signals were detected in the anatomical location of brain at 0.5, 1, 3, and 6 h after injection. The data further suggested that the BBB-penetrating ability of AptB1 is irrelevant to the BBB disruption introduced by CNS cancer metastasis.

### Detection of AptB1 Sequences in the Cerebrospinal Fluid (CSF)

**Figure 3:**
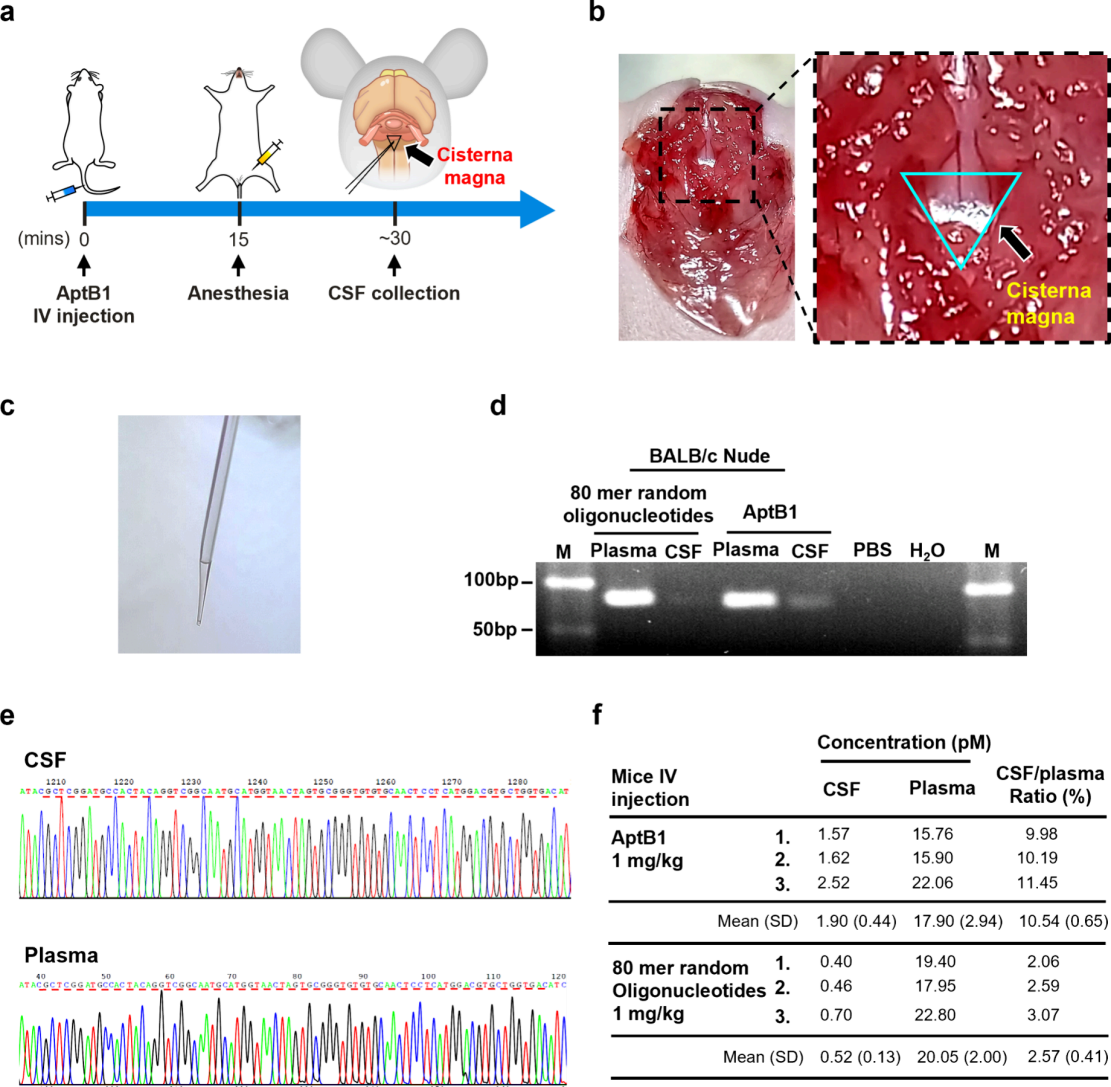
Detection of AptB1 in the CSF. (a) The CSF was sampled directly from the cisterna magna 30 min after AptB1 injection through the tail vein. (b) Gross picture of mouse cisterna magna (blue triangle). (c) The CSF was sampled from the cisterna magna with a capillary. (d) The AptB1 was amplified from the CSF and the plasma. The PCR cycle number was 20. (e) Accuracy of the amplified AptB1 sequences was confirmed by Sanger sequencing. (f) Concentrations of the CSF and the plasma AptB1 were determined by qPCR. SD: standard deviation.

To further confirm that the AptB1 signals detected via the IVIS and by confocal microscopy were from the aptamers located in the ventricular space instead of CNS microcirculation, the CSF was sampled directly from the cisterna magna with glass capillary tubes. In brief, the AptB1 or the 80-nucleotide-long ssDNA molecules (80-mer random oligonucleotides) were intravenously (IV) injected through the tail vein, and the CSF was collected approximately 30 min after the injection (Figure 3a–c). To detect AptB1 sequences, the CSF was PCR amplified with primers specific to our aptamer library. As shown in the agarose gel electrophoresis, the AptB1 amplicons were detected in both the plasma and the CSF sampled from the mouse administered with AptB1 (Figure 3d). The sequence accuracy was verified by Sanger sequencing (Figure 3e). Next, the percent of penetration across the BBB was measured by qPCR. The AptB1 or the 80-mer oligonucleotide random sequences (control), 1 mg/kg each, were IV injected through the tail vein. The plasma and the CSF were sampled 30 min after drug administration as already mentioned, and the samples were subjected to qPCR for quantification. As shown in the Figure 3f, the CSF to plasma ratio for the AptB1 was 10.54%, and that for the oligonucleotide random sequences was 2.57%. The data further supported the BBB-penetrating ability of AptB1.

### AptBCis1, an Aptamer–Cisplatin Conjugate, Showed Antitumor Effects in Lung Cancer LM Diseases

**Figure 4:**
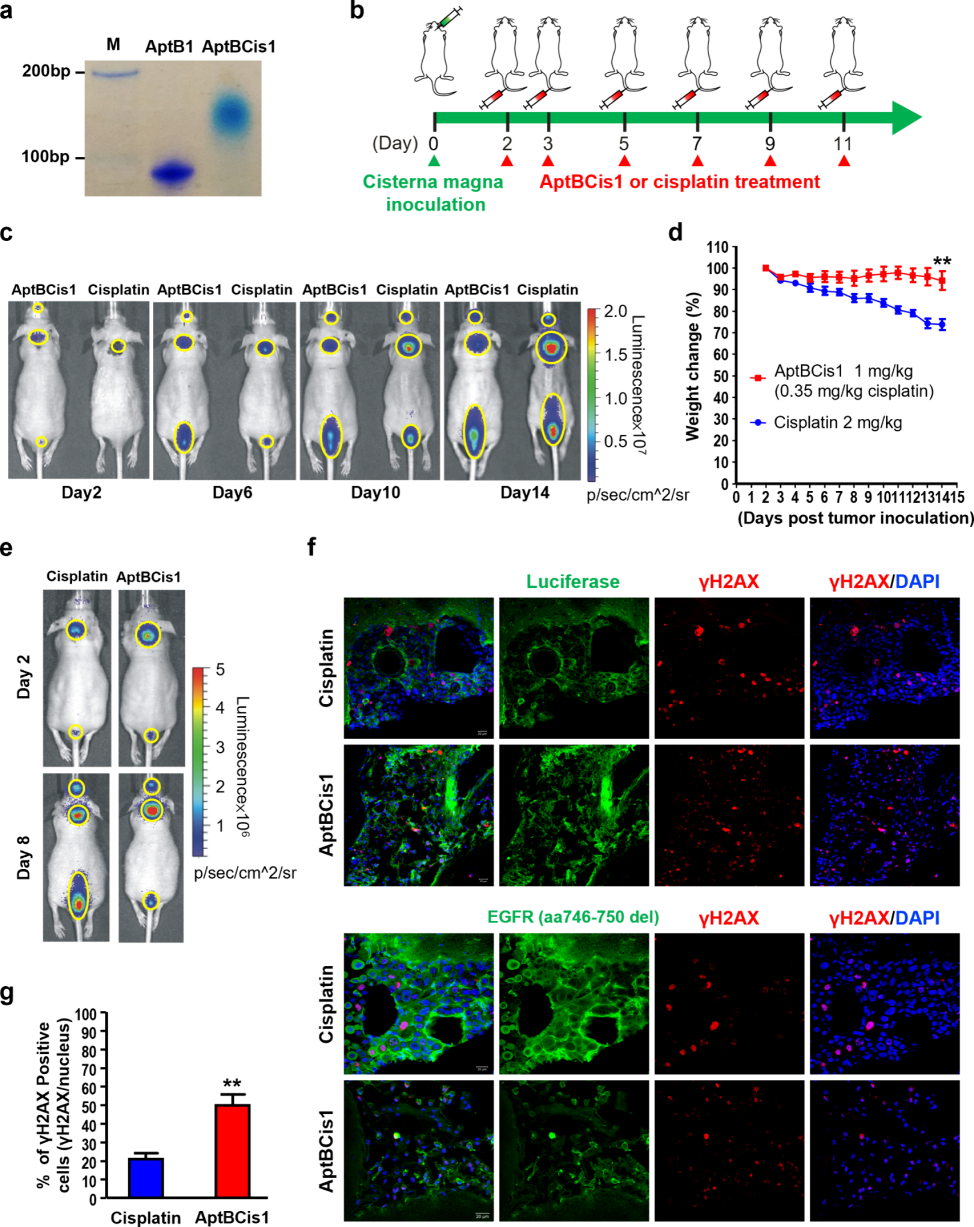
AptBCis1, an aptamer–cisplatin conjugate, showed antitumor effects in lung cancer LM diseases. (a) The native polyacrylamide gel electrophoresis result showed successful conjugation of the AptB1 and the cisplatin. (b) The scheme illustrated the timeline of tumor cell inoculation and IV drug treatment (AptBCis1 or cisplatin). (c) The mice were monitored by IVIS; BLI signal intensity implicated corresponding tumor burden. Mice in the cisplatin group showed stronger BLI signals than mice in the AptBCis1 group on Day 14 post tumor inoculation (*n* = 8 in each group). The lesions are outlined with yellow circles. (d) Significant body weight reduction in the cisplatin group (**P < 0.01). Formulation of body weight change: (Day X/Day 2) %. (e) The IVIS images were taken at Day 2 and Day 8 post tumor inoculation; cisplatin or AptBCis1 was given on Days 2, 3, 5, and 7 post tumor inoculation. The mice were sacrificed on Day 8, and the brains were subjected to immunofluorescent studies. The lesions are outlined with yellow circles. (f) The confocal microscopy images showed better preserved tumor cell contours (green; upper panel: luciferase; lower panel: EGFR) and lower percentage of γH2AX-positive cells (red) in the cisplatin group. (g) A lower percentage of γH2AX-positive cells was observed in the cisplatin group. A total of 1200 or 450 cells, respectively, was analyzed in the cisplatin or the AptBCis1 group. Asterisks denote statistically significant differences. **P < 0.01 (unpaired *t* test).

To develop an AptB1-chemotherapeutic agent conjugate for lung cancer LM diseases, we conjugated cisplatin to AptB1, forming AptBCis1. The successful conjugation was demonstrated by 16% nondenaturing polyacrylamide gel electrophoresis—AptBCis1 had a slower moving rate owing to its greater positive charges contributed by the cisplatin (Figure 4a). Next, AptBCis1 was subjected to inductively coupled plasma optical emission spectrometry analyses. The results showed that each AptB1 sequence segment contained approximately 27 platinum molecules.

We then examined the tumor suppressive effects of AptBCis1 using a lung cancer LM orthotopic model. In brief, PC9 lung cancer cells were inoculated directly into the cisterna magna, and AptBCis1 (1 mg/kg, with equivalent cisplatin concentration of 0.35 mg/kg) or cisplatin (2 mg/kg) was IV administered via tail vein at Day 2, 3, 5, 7, 9, and 11 post tumor inoculation (Figure 4b). Tumor growth was monitored via IVIS, and mouse body weight was measured daily. As shown in Figure 4c, BLI signals emitted from the cancer cells were much stronger in the cisplatin group than in the AptBCis1 group, suggesting better tumor suppressive effects with AptBCis1 than with cisplatin. In line with the IVIS data, reduction of mouse body weight was modest in the AptBCis1 group at Day 14 post tumor inoculation, while that was significant in the cisplatin group (Figure 4d), supporting the effect of AptBCis1 on tumor suppression.

To ensure that the tumor suppression was attributed by the cisplatin-induced cytotoxicity, the AptBCis1 or the cisplatin-treated mice were sacrificed on Day 8 post tumor inoculation, and the brains were examined by immunofluorescent studies (Figure 4e). In brief, cryosections of the brain were costained with γH2AX-specific antibody, and luciferase or EGFR (aa746-750del)-specific antibody. The γH2AX is a marker for cisplatin-induced DNA double-strand break;^13^ the luciferase expression indicates tumor cells in our system, and the EGFR (aa746-750del) antibody specifically targets EGFR Del19-expressing PC9 cells. As shown in Figure 4f, tumor cells in the AptBCis1 group highlighted by the luciferase or the EGFR signals showed discohesiveness of the cells with fragmented cell mass, indicating extensive cell death; in contrast, tumor cells in the cisplatin group had a complete plasma membrane, indicating viability of the cells. Moreover, the γH2AX signals were more extensively distributed across tumor cells in the AptBCis1 group than in the cisplatin group, suggesting a higher degree of DNA double-strand break induced by the AptBCis1 treatment (Figure 4f,g). Taken together, our data suggested better tumor suppressive effects of AptBCis1 than cisplatin on lung cancer LM diseases.

### AptBCis1 Inhibits Lung Cancer Growth at Lower Cisplatin Concentrations

The LM mouse data showed better tumor suppressive effects with AptBCis1 at a lower equivalent systemic cisplatin concentration (0.35 mg/kg) than with cisplatin (2 mg/kg). It is, therefore, of particular importance to determine the CSF platinum concentration in these two scenarios.

**Figure 5:**
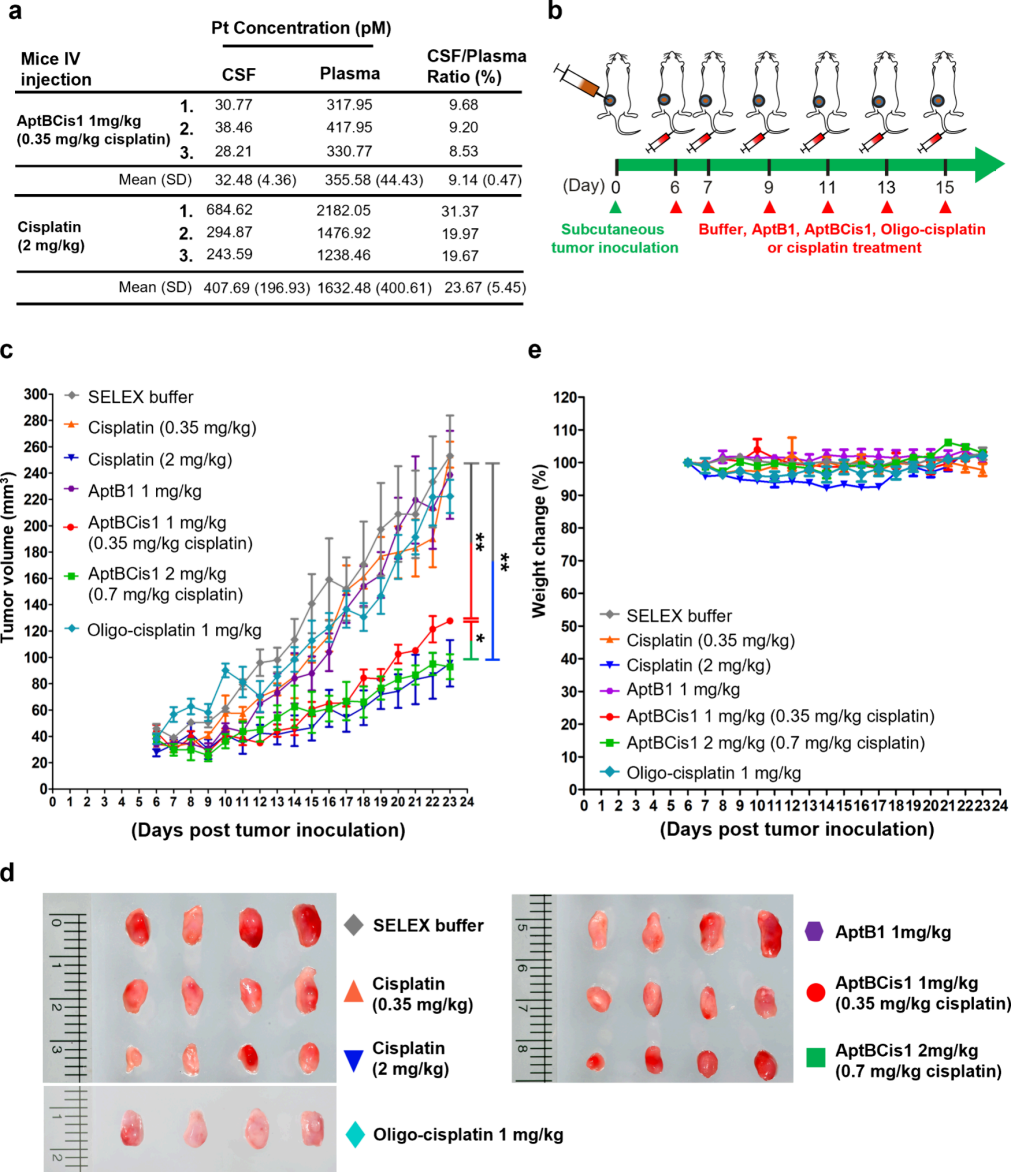
AptBCis1 suppressed tumor growth at lower platinum concentrations. (a) The plasma and the CSF platinum concentrations were measured by ICP-MS. (b) The scheme illustrated the timeline of subcutaneous tumor cell inoculation and drug treatment in the lung cancer subcutaneous xenograft mouse model. The SELEX buffer, AptB1, AptBCis1, oligo-cisplatin or cisplatin was given via tail vein at Day 6, 7, 9, 11, 13, and 15 post tumor inoculation (*n* = 4 for each group). (c) Tumor size was measured daily, and the mice were sacrificed on Day 23. (d) Tumor gross pictures. (e) Body weight reduction was more obvious in the cisplatin 2 mg/kg group. Asterisks denote statistically significant differences. *P < 0.05, **P < 0.01 (unpaired *t* test).

In brief, CSF from the tumor-naïve mouse was directly sampled at 30 min after tail vein injection, either with AptBCis1 at 1 mg/kg or with cisplatin at 2 mg/kg. The CSF and paired plasma specimens were subjected to inductively coupled plasma mass spectrometry (ICP-MS) for platinum concentration determination. The ICP-MS results showed that the CSF platinum concentration with AptBCis1 (1 mg/kg) treatment was one tenth of that with cisplatin (2 mg/kg) treatment (Figure 5a). Moreover, the ICP-MS data suggested a 10% or a 20% CSF to plasma ratio, respectively, with AptBCis1 or cisplatin treatment. The analysis with agarose gel electrophoresis on the PCR-amplified samples further suggested a 10% CSF to plasma ratio of AptBCis1 (Figure S2). With better tumor suppressive effects at lower CSF platinum concentrations observed in the AptBCis1 treatment group, the data implicated that AptBCis1 exerted its antitumor effect beyond the scope of total platinum concentration.

To prove this hypothesis, we examined antitumor effects of AptBCis1 using lung cancer subcutaneous xenograft mouse models. In brief, PC9 lung cancer cells were inoculated onto the back of the BALB/c nude mice. The SELEX buffer control and different treatments, respectively cisplatin 0.35 mg/kg, cisplatin 2 mg/kg, AptB1 1 mg/kg, AptBCis1 1 mg/kg (0.35 mg/kg cisplatin), AptBCis1 2 mg/kg (0.7 mg/kg cisplatin), or oligo-cisplatin 1 mg/kg, were given via tail vein on days 6, 7, 9, 11, 14, and 15 post tumor inoculation (Figure 5b). The results showed that AptBCis1 1 mg/kg (0.35 mg/kg cisplatin) had greater tumor suppressive effects than cisplatin 0.35 mg/kg, and its effect was only slightly inferior to that of high-dose cisplatin (2 mg/kg). Of note, AptBCis1 2 mg/kg (0.7 mg/kg cisplatin) showed tumor suppressive effects identical to those of high-dose cisplatin (2 mg/kg). On the other hand, treatment with SELEX buffer, AptB1 1 mg/kg, oligo-cisplatin 1 mg/kg, or cisplatin 0.35 mg/kg exhibited no tumor inhibitory effects (Figure 5c,d). Consistent with the LM orthotopic mouse model data, the results of the subcutaneous xenograft mouse model also showed that AptBCis1 exhibited better tumor suppressive effects at equivalent cisplatin concentrations lower than those of cisplatin alone. The results once again strengthen the hypothesis that AptBCis1 exerted its antitumor effects beyond the issue of total platinum concentration.

Moreover, while mouse body weight reduction was modest in the AptBCis1 1 or 2 mg/kg treatment groups, a 10% body weight loss was observed in the cisplatin 2 mg/kg treatment group (Figure 5e). The data suggested the safety of AptBCis1 at its effective dose.

### AptB1 Binds to EAAT2, Nucleolin, and YB-1 Proteins

**Figure 6:**
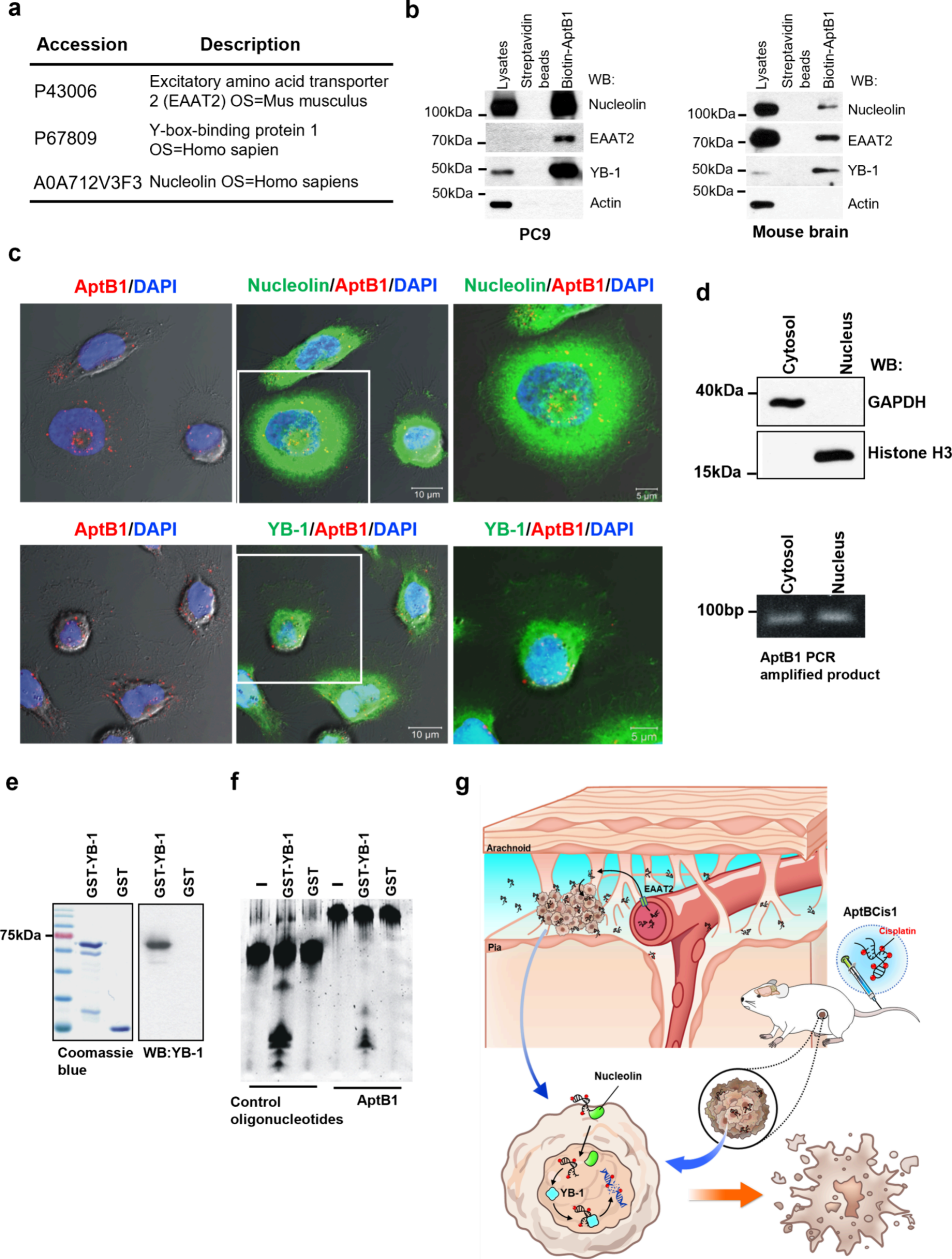
AptB1 interacting with EAAT2, Nucleolin, and YB-1. (a) Results of AptB1-AP/MS study revealed three candidate AptB1-interacting proteins: EAAT2, YB-1, and Nucleolin. (b) AP-immunoblots verified the interaction between AptB1 and EAAT2, Nucleolin, as well as YB-1 in the PC9 cells and in the mouse brain. (c) The confocal microscopy images showed colocalization (yellow) of AptB1 (red) and Nucleolin (green, upper panel) or YB-1 (green, lower panel) in the PC9 cells. (d) AptB1-treated cells were fractionated into cytosol and nucleus fractions. GAPDH is a cytosolic marker, and Histone H3 is a nucleus marker. The AptB1 sequences were successfully amplified in both cellular fractions. (e) The Coomassie Blue stain and the immunoblots showed the purified GST and GST-YB-1 proteins. (f) The YB-1 exonuclease assay results supported the role of YB-1 as an exonuclease for AptB1. (g) Scheme illustrating the proposed mechanism of AptBCis1 as therapeutics for lung cancer with and without LM.

To elucidate the mechanisms, we next investigated the AptB1-interacting proteins. Aptoprecipitation (AP, aptamer-based protein precipitation) with AptB1 was performed using total cell lysates prepared from mouse brains or PC9 lung cancer cells. Mass spectrometry (MS) analyses for the aptoprecipitants revealed three candidate proteins: excitatory amino acid transporter 2 (EAAT2), Y-Box binding protein 1 (YB-1), and Nucleolin (Figure 6a).

The results were then verified with AptB1 AP-immunoblots. The mouse brain tissue and the PC9 cell aptoprecipitants were immunoblotted with the EAAT2, YB-1, or Nucleolin antibody. Antibody specificity was confirmed by the immunoblots on total cell lysates prepared from the mouse endothelial (bEnd3) or the human lung cancer (PC9) cells with or without SiRNA treatment, respectively, SiEAAT2, SiYB-1, or SiNucleolin (Figure S3a). The results showed that signals for EAAT2, YB-1, and Nucleolin were detected both in the mouse brain and in the PC9 aptoprecipitants but with different signal intensities. The signal for EAAT2 was the strongest in the mouse brain but the weakest in PC9. The signal for Nucleolin went opposite in these two aptoprecipitants, being the strongest in PC9 but the weakest in the mouse brain (Figure 6b). Knockdown of EAAT2 in bEnd3 cells led to a reduced cellular uptake of AptB1 (Figure S3b). Confocal microscopy images further visualized the aptamer–protein interactions. As shown in the Figure 6c, signals for AptB1 (red) and Nucleolin (green; upper panel) as well as AptB1 (red) and YB-1 (green, lower panel) colocalized (yellow) in the PC9 cells, both in the cytoplasm and in the nucleus.

To further confirm the existence of AptB1 in the cell nucleus, AptB1-treated PC9 cells were separated into the cytosol and the nucleus fractions. DNAs were independently extracted from these two fractions, and the AptB1 sequences were amplified with AptB1-specific primers. The successful amplification of AptB1 from the nucleus fraction supported the entrance of AptB1 into cell nucleus (Figure 6d and Figure S3c).

Cisplatin interacts with DNA and forms a covalent adduct with purine DNA bases and platinum compound.^6^ Therefore, the AptB1 DNA backbone must be digested before cisplatin can be released from AptBCis1, so as to exert its cytotoxic effect on targeted cancer cells. Prior studies suggested the role of YB-1 as an exonuclease.^14^ To test if YB-1 served as the exonuclease for AptB1, the 5′-FAM-labeled AptB1 or the control oligonucleotides were incubated with the purified GST-YB-1 or the GST proteins (Figure 6e). As shown in Figure 6f, the AptB1 and control oligonucleotides were both digested by the GST-YB-1 proteins, resulting in smears shown on the 22% polyacrylamide gel. The data supported the role of YB-1 as an exonuclease for AptBCis1.

Taken together, we showed that AptB1 binds to EAAT2, Nucleolin, and YB-1. The binding with EAAT2 could contribute to the BBB-penetrating ability of AptB1. The interaction with Nucleolin may explain the efficient nucleus delivery of AptBCis1. The binding with YB-1 in turn leads to the digestion of the AptB1 DNA backbone, facilitating the release of cisplatin from AptBCis1. All these together constituted the promising tumor suppressive effects of AptBCis1 at lower cisplatin concentrations, in both lung cancer LM orthotopic and subcutaneous xenograft mouse models (Figure 6g).

### Discussion

Leptomeningeal carcinomatosis remains a clinical challenge, with limited available therapeutic options. This is partly attributed to the biological nature of BBB, which restrains the entry and actively pumps out therapeutic agents from the CNS.^1−3^ In the current study, we reported a DNA therapeutics, AptBCis1. The AptBCis1 is a cisplatin-conjugated and BBB-penetrating DNA aptamer that targets cancer cells. Its backbone, AptB1, was identified via *in vivo* SELEX using a lung cancer LM orthotopic mouse model. The AptB1 binds to EAAT2, Nucleolin, and YB-1 proteins.

EAAT2 is a Na^+^-dependent glutamate transporter located on the BBB.^15^ Nucleolin is a multifunctional RNA-binding protein located in the nucleolus, nucleoplasm, cytoplasm, and cell membrane. Of note, upregulation of membranous Nucleolin is common in cancer, executing functions related to cancer progression, such as mediating nuclear translocation of cancer-associated proteins.^16−19^ YB-1 is a transcription factor located in the cytoplasm and nucleus. It preferentially binds to single-stranded nucleic acids and exhibits exonuclease activities.^14^ The interactions with EAAT2 and Nucleolin could contribute to the BBB-penetrating, the cancer-targeting, and the nuclear-translocating abilities of the AptB1/AptBCis1. The interaction with YB-1 could contribute to the degradation of the AptB1 DNA backbone and the release of cisplatin. All these together constituted the antitumor activity of AptBCis1, both in the CNS and in the peripheral tissue compartments. As shown in our study, AptBCis1 exhibited promising tumor suppressive effects at lower cisplatin concentrations in both the lung cancer LM orthotopic and subcutaneous xenograft mouse models.

For lung cancer with druggable driver mutations, several CNS-effective small molecule inhibitors have been successfully developed, such as osimertinib, alectinib, and lorlatinib.^1,2,4,5^ Nevertheless, the emergence of resistance mutations and/or the activation of alternative pathways that nearly always comes along with each line of therapy eventually leads to treatment failure and disease progression. Therefore, chemotherapy continues to serve as a salvage therapy. Moreover, for lung cancer without druggable drivers, chemotherapy with or without immune checkpoint inhibitors is the current standard of care.^4,5^ In other words, platinum-based drugs still play irreplaceable roles in lung cancer therapy. The drugs, however, are notorious for neurotoxicity and are subjected to a complex set of resistance mechanisms.^6^ The former restricts their cumulative dose threshold, and the latter compromises their true effective intracellular concentrations. Therefore, a smart delivery system that simultaneously augments intracancer platinum effects and minimizes its systemic side effects would be valuable. AptBCis1, an aptamer–cisplatin conjugate, is a therapeutics of this kind.

The guiding molecule for delivery is AptB1, a DNA aptamer identified through *in vivo* SELEX using a lung cancer LM orthotopic mouse model. Different from *in vitro* aptamer selection methodologies, this *in vivo* platform took advantage of biological environments that facilitate the identification of candidate aptamers with predetermined functions—BBB penetration and cancer targeting. For example, the biological complexity of the cancer-bearing mouse by itself helped reduce the SELEX round required, which in turn preserved the diversity of sequences at each selection round, so as to facilitate the identification of aptamers with the given biological functions.

A major limitation for DNA drugs is their susceptibility to degradation with short *in vivo* half-life.^9−11^ In our system, the selection process was carried out in a living animal system that was full of DNA nucleases. Therefore, the sequences detected in the final selection round are relatively stable *in vivo*, because of sequence evolution through selection. Furthermore, the biodistribution study of AptBCis1 showed strong signals in bladder, kidney, and liver (Figure S4), which are the organs reported to be involved in aptamer metabolism and excretion. Interestingly, such a biodistribution pattern was similar to that of cisplatin.^20,21^ In addition, recent studies further showed feasibility of aptamer therapeutics in neurodegenerative disorders, such as Parkinson’s disease and Tauopathies, and illustrated its pharmacokinetics in human subjects.^22−24^ Taken together, these lines of evidence suggested the translational potential of aptamer therapeutics in overcoming delivery barriers in human diseases, such as the BBB. Further preclinical investigations on pharmacodynamics and pharmacokinetics of AptBCis1 are warranted for its clinical translation.

## Conclusions

In summary, we reported here a cisplatin-conjugated DNA aptamer therapeutics, AptBCis1. AptBCis1 is effective in lung cancer with leptomeningeal carcinomatosis at lower cisplatin concentrations. The results suggested the translational potential of AptBCis1 in lung cancer with LM, and in cancers for which platinum-based chemotherapy remains as the standard of care.

## Methods and Experiments

### Animal Study

All mouse experiments were performed in BALB/c nude mice of matching age (6 weeks; weight ∼20 g). The mice were obtained from the National Laboratory Animal Center (Taipei, Taiwan). The experiments were approved by the Department of Animal Care, Institute of Biomedical Sciences, Academia Sinica, Taiwan (IACUC approval number: IBMS-CRC100-P02).

### Cells and Cell Culture

The human lung cancer cell line CL1-5 was established in our laboratory,^25^ and PC9 cells were obtained from a collaborative laboratory in the National Taiwan University Hospital. CL1-5 and PC9 cells were cultured in RPMI-1640 medium (Invitrogen) supplemented with 10% fetal bovine serum (FBS) and 100 μg/mL Primocin (InvivoGen, USA). Cells used for experiments were all within 10 passages after thawing. The mouse endothelial cell line, bEnd.3, was obtained from the Bioresource Collection and Research Center Taiwan and cultured in high glucose Dulbecco’s modified Eagle’s medium (Life Technologies, Grand Island, NY), supplemented with 10% FBS, HEPEs, 100 U/mL penicillin, and 100 μg/mL streptomycin. Stably transfected, pooled clones were maintained in a medium supplemented with 2 μg/mL puromycin (InvivoGen) or 400 μg/mL G418 (InvivoGen). Mycoplasma testing was performed regularly using a PlasmoTest Kit (InvivoGen).

### Chemicals, Oligonucleotides, siRNA, and Antibodies

All chemicals were purchased from Sigma-Aldrich. Aptamer libraries or modified aptamers were synthesized by Integrated DNA Technologies (Coralville, IA, USA) or Purigo Biotech (Taipei, Taiwan). The synthetic single-stranded DNA library was composed of 80-nucleotide-long single-stranded DNAs with 40 random sequences flanked by primer sequences, 5′-ACGCTCGGATGCCACTACAG[N]_40_CTCATGGACGTGCTGGTGAC, N = A, T, G, C. Primers for mouse mRNA or genomic DNA amplification were as follows F′, CATCACTGCCACCCAGAAGACTG; R′, ATGCCAGTGAGCTTCCCGTTCAG. Anti-luciferase antibody (sc-74548), EAAT2 siRNA (sc-35256), Nucleolin siRNA (sc-29230), YB-1 siRNA (sc-38634), and GAPDH antibody (sc-32233) were purchased from Santa Cruz. γH2AX (9718), YB-1 (4202), and EAAT2 (20848) antibodies were purchased from Cell Signaling. Human EGFR (aa 746-750 deletion; EGFRdel19) antibody (MAB8336) was purchased from R&D systems. Nucleolin antibody (ab22758) was purchased from Abcam. Histone H3 antibody (GTX1222148) was purchased from GeneTex.

### Leptomeningeal Carcinomatosis (LM) Orthotopic Mouse Models

BALB/c nude mice at 6 weeks of age were used for the LM orthotopic mouse model establishment. Stable cells used included luciferase-expressing PC9 or CL1-5 cells. To generate an LM orthotopic mouse model for *in vivo* SELEX or for AptBCis1 efficacy study, respectively, 1 × 10^6^ or 3 × 10^5^ cells were resuspended in 10 μL PBS (137 mM NaCl, 2.7 mM KCl, 10 mM Na_2_HPO_4_·2H_2_O, 2 mM KH_2_PO_4_, pH 7.4) and were inoculated directly into the cisterna magna of the anesthetized recipient mice.^26^ Tumor burden was monitored via an *in vivo* imaging system (IVIS, Xenogen Caliper IVIS spectrum, USA), and mouse body weight was measured daily.

### Subcutaneous Xenograft Mouse Models

For the subcutaneous xenograft mouse model, 5 × 10^5^ PC9 cells were resuspended in 10 μL of PBS and inoculated onto the right flank of BALB/c nude mice at 6 weeks of age. Tumor size and mouse body weight were measured daily. Volume was calculated as follows: length × (width)^2^ × 0.51.

### Immunohistochemistry Study

To ensure the successful establishment of LM orthotopic mouse models, the mice were euthanized at Day 6 after tumor inoculation, upon which strong bioluminescent (BLI) signals were detected by IVIS over the anatomical locations of brain and spinal cord. Transcardiac perfusion with PBS and 4% paraformaldehyde was performed prior to organ isolation. The isolated brain and spine were fixed in 4% paraformaldehyde overnight at 4 °C. The tissues were then embedded in paraffin and built into tissue blocks. For the histology study, the tissue slides were rehydrated and blocked with PBS containing 10% normal goat serum, 2% BSA, and 0.2% Triton X-100 (PBST). To detect tumor cells, luciferase antibody (sc-74548, Santa Cruz) was prepared with PBST containing 10% normal goat serum and 1% BSA. After overnight incubation at 4 °C, the samples were washed and stained with HRP-conjugated secondary antibody (PK-6102, Vector).

### Immunofluorescence Study

Immunofluorescence (IF) studies were performed on tissue sections to detect LM tumor cells, aptamers, and several protein markers. After sacrification, transcardiac perfusion with PBS and 4% paraformaldehyde was performed prior to organ isolation. The isolated brain was fixed in 4% paraformaldehyde at 4 °C overnight and was transferred to a 30% sucrose bath at 4 °C for another 24 h. The samples were embedded in the OCT compound and made into cryosections. For the IF staining, the samples were blocked with PBST containing 10% normal goat serum and 2% BSA. Hybridization with luciferase, EGFRdel19, γH2AX, Nucleolin, or YB-1 antibodies was carried out overnight at 4 °C. The secondary antibodies used included anti-FITC and anti-Cy3. Images were acquired using a Zeiss LSM700 confocal microscope (Carl Zeiss Microimage, Thornwood, NY).

### *In Vivo* SELEX

For *in vivo* SELEX, 2 × 10^15^ single-stranded DNA (ssDNA) oligonucleotides were dissolved in the SELEX buffer (140 mM NaCl, 4 mM KCl, 1 mM MgCl_2_, 1 mM CaCl_2_, and 40 mM HEPES, pH 7.4). The ssDNA oligonucleotide library was intraperitoneally injected into the LM mouse, after which tumor signals were detected in the anatomical location of brain by IVIS (Xenogen Caliper IVIS spectrum, USA) at Day 6 post tumor inoculation. The mouse was anesthetized and was perfused with the SELEX buffer to remove the unbound oligonucleotides at 6 h post ssDNA library administration. The collected brain tissue was snap-frozen by liquid nitrogen followed by proteinase K digestion at 55 °C overnight. The DNA was extracted with a Gentra Puregene Tissue Kit (Qiagen); the extracted DNAs served as the sample for oligonucleotide sequence amplification. The amplified oligonucleotide sequences were then subjected to single-stranded isolation and refolding as previously described^27−29^ and were intraperitoneally injected into the LM mouse for subsequent SELEX rounds. After the fourth SELEX round, the amplified oligonucleotide sequences were ligated into the CloneJet vector (Thermo Fisher) for colony PCR. The amplicon sequences were determined by Sanger sequencing (ABI3730, Applied Biosciences). Grouping of the identified aptamers was performed based on the presence of probable G-quadruplex structures or not, as predicted by QGRS Mapper (https://bioinformatics.ramapo.edu/QGRS/index.php).

### Cerebrospinal Fluid (CSF) and Plasma Collection

Plasma was sampled via a cardiac puncture. Blood was centrifuged immediately after sampling and the supernatant was transferred to sterile Eppendorf tubes. CSF was sampled through surgical procedures. In brief, the mouse was anesthetized; the skin, the subcutaneous tissue, and the muscle were dissected from the posterior neck to expose cisterna magna with the assistance of microscopy and micromanipulator (M650, Wild Heerbrugg). CSF was collected via direct dura puncture with capillaries. The samples were stored in a −80 °C freezer until use.

### AptB1 and Platinum Concentration Measurement

Concentrations of CSF and plasma AptB1 were measured via quantitative PCR (qPCR). For quantification, a standard curve for AptB1 was established with 80-mer oligonucleotides: concentrations of the 80-mer oligonucleotides were serially diluted from 100 to 0.01 PM to constitute the formula *Y* = −0.2982*X* + 5.3802, *R* = 0.9997. Concentrations of AptB1 were measured accordingly. Concentrations of CSF or the plasma platinum were determined by inductively coupled plasma mass spectrometry (ICP-MS) (Thermo Fisher Scientific) analysis.

### Aptamer–Cisplatin Conjugation

Cisplatin powder was purchased from Sigma, and the drug was dissolved in double-distilled water (ddH_2_O) at a concentration of 1 mg/mL. The aptamer was denatured at 95 °C, cooled to 4 °C, and refolded at 37 °C in the SELEX buffer prior to cisplatin conjugation. The conjugation was carried out per protocol.^30,31^ The success of conjugation was confirmed by gel electrophoresis (16% PAA nondenaturing gel; monoacrylamide to bis(acrylamide) ratio of 19:1), and signal visualization was made with STAINS-ALL (Sigma-Aldrich). The platinated aptamers were further analyzed with inductively coupled plasma optical emission spectrometry (ICP-OES) (Varian 720-ES, Agilent Technologies) to measure the quantity of conjugated platinum. The platinated aptamers were dissolved in the SELEX buffer and were stored at −20 °C until use.

### AptBCis1 Treatment in the LM Orthotopic Mouse Model

AptBCis1 or cisplatin was injected through the tail vein at Days 2, 3, 5, 7, 9, and 11 post tumor cell inoculation. The dosage of AptBCis1 used was 1 mg/kg (approximately equal to cisplatin 0.35 mg/kg), and that of cisplatin was 2 mg/kg. Tumor burden was monitored by IVIS (Xenogen Caliper IVIS spectrum, USA), and mouse body weight was measured daily. Each group consisted of 8 mice.

### AptBCis1 Treatment in the Subcutaneous Xenograft Mouse Model

BALB/c nude mice inoculated with PC9 cells (5 × 10^5^) at the flank were divided into 6 groups on Day 6 post inoculation, at which time point the tumor volume reached about 30–40 mm^3^. The drugs, respectively (1) SELEX buffer, (2) cisplatin 0.35 mg/kg, (3) cisplatin 2 mg/kg, (4) AptB1 1 mg/kg, (5) AptBCis1 1 mg/kg (0.35 mg/kg cisplatin), (6) AptBCis1 2 mg/kg (0.7 mg/kg cisplatin), and (7) oligo-cisplatin 1 mg/kg, were given via tail vein. Tumor volume and mouse body weight were measured daily. Each group consisted of 4 mice.

### Aptoprecipitation

The cells or the homogenized mouse brain (Dounce Tissue Grinder; Wheaton) was prepared in lysis buffer (50 mM Tris, pH 7.5, 150 mM NaCl, 5 mM MgCl_2_, 1 mM EDTA, 5% glycerol, and 1% NP-40) containing protease inhibitors (Thermo Fisher). The biotinylated aptamer was conjugated with Streptavidin in 1 × SSC buffer at 4 °C for 3 h and was then washed with lysis buffer. The biotinylated aptamer or the Streptavidin-Sepharose agarose beads (Amersham pharmacia) were incubated with cell lysates overnight at 4 °C, and the samples were washed 4 times with detergent-free lysis buffer. The aptoprecipitants were then denatured at 95 °C in the SDS sample buffer and were subjected to immunoblots for analysis.

### Exonuclease Assay

The control oligonucleotide 5′-FAM-TCGATCGGGGCGGGGCGATCGGGGCGGGGCGA (20 ng) or the 5′-FAM AptB1 aptamer (20 ng) was mixed with purified GST or GST-YB-1 proteins (400 ng each) and was incubated in the reaction buffer (50 mM Tris, pH7.5, 10 mM MgCl_2_) at 35 °C for 4 h. After brief centrifugation, the reaction products were separated by 22% acrylamide gel with 8 M urea. The gel was then scanned by a Typhoon 9410 (GE).

## Data Availability

All data are available in the main text or the online Supporting Information.

## Supporting Information Available

Aptamer sequences identified by *in vivo* SELEX, IVIS images of the mice studied, AptBCis1 BBB penetration rate, results of antibody specificity, effects of EAAT2 knockdown on AptB1 uptake by bEnd3 cells, Sanger sequencing of the AptB1 sequence, and biodistribution of AptBCis1 in nude mice (PDF)
