# Supplementary material for: AptBCis1, An Aptamer–Cisplatin Conjugate, Is Effective in Lung Cancer Leptomeningeal Carcinomatosis
Source: ACS Nano. 2024 Oct 3;18(41):27905–16. doi: 10.1021/acsnano.4c04680 (PMC11483943; doi:10.1021/acsnano.4c04680)
Supplement: Supplementary file 1 — nn4c04680_si_001.pdf [file nn4c04680_si_001.pdf]

Supporting Information

AptBCis1, An Aptamer-Cisplatin Conjugate, is Effective in Lung Cancer Leptomeningeal Carcinomatosis

Bo-Tsang Huang, Wei-Yun Lai, Chen-Lin Yeh, Yi-Ting Tseng, Konan Peck, Pan-Chyr Yang, Emily Pei-Ying Lin

Table S1. Aptamer sequences identified by *in vivo* SELEX

Figure S1. IVIS images of the mice studied

Figure S2. AptBCis1 BBB penetration rate

Figure S3. Results of antibody specificity, effects of EAAT2 knockdown on AptB1 uptake by bEnd3 cells, and Sanger sequencing of the AptB1 sequence

Figure S4. The biodistribution of AptBCis1 in nude mice

Table S1. Aptamer sequences identified by *in vivo* SELEX

ID		ΔG(kcal/mol)
AptB1	ACGCTCGGATGCCACTACAGGTCGGCAATGCATGGTAACTAGTGCGGGTGTGTGCAACTCCTCATGGACGTGCTGGTGAC	-16.72
AptB2	ACGCTCGGATGCCACTACAGGGGGTAGGTTCGCCGGGTCCAAATGCCTATTATCATCCAACTCATGGACGTGCTGGTGAC	-14.86
AptB3	ACGCTCGGATGCCACTACAGCTCCATACGTATCTCCACCATTCCTTGGGGATTTATAAGTCTCATGGACGTGCTGGTGAC	-17.32
AptB4	ACGCTCGGATGCCACTACAGATGTTCCAACTATAGTTGGGGTCAAATCTTCCCAATGGTGCTCATGGACGTGCTGGTGAC	-13.41
AptB5	ACGCTCGGATGCCACTACAGATTTTAAGTAACCCGTTGCACCCCACAACACCGTGGCGATCTCATGGACGTGCTGGTGAC	-14.63
AptB6	ACGCTCGGATGCCACTACAGAAAAGCTCAAGCCTCAATGAATTAACTGACGCGTCGTTGTCTCATGGACGTGCTGGTGAC	-14.62
AptB7	ACGCTCGGATGCCACTACAGTTGGTAACAGGCTTAATTGAGTATGCCTTTTCTGGTGAAACTCATGGACGTGCTGGTGAC	-15.24
AptB8	ACGCTCGGATGCCACTACAGCTGCTCTAAGGCTTATACGTATACCTCCTTTACATTTGCACTCATGGACGTGCTGGTGAC	-12.43
AptB9	ACGCTCGGATGCCACTACAGACATGTTTTCTCTGTTCATCTATGGTTTGGGACTCTTTTCTCATGGACGTGCTGGTGAC	-10.11

a

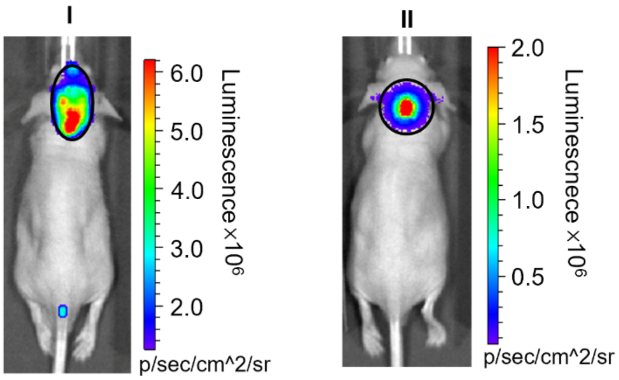

b

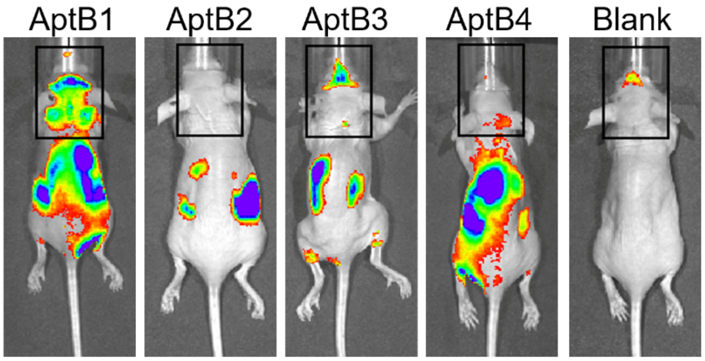

ID	Avg Radiance ($p/s/cm^2/sr$)
**AptB1**	2.20E+08
**AptB2**	1.262E+08
**AptB3**	1.575E+08
**AptB4**	1.493E+08
**Blank**	1.227E+08

c

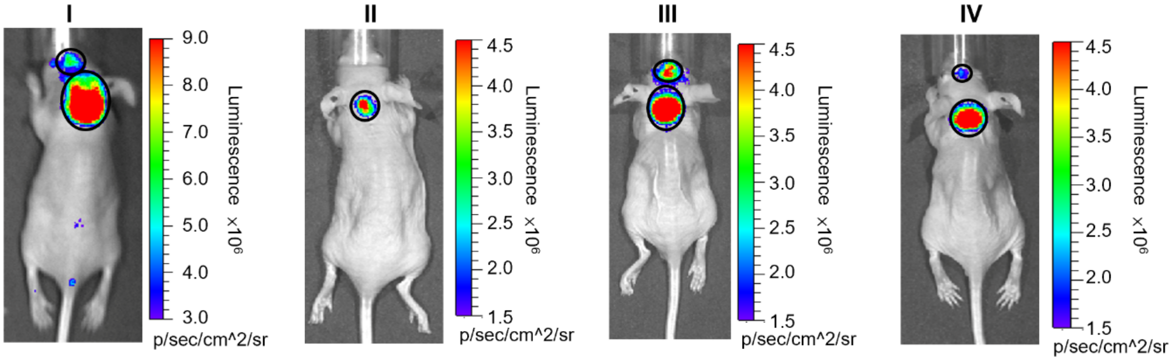

d

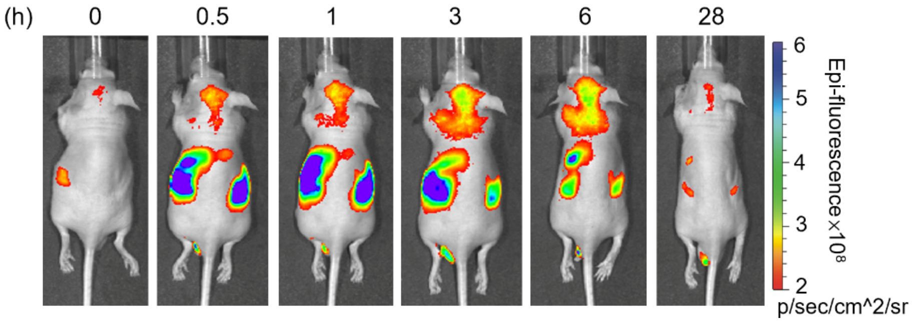

Figure S1. IVIS images of the mice studied. (A) BLI signals emitted from the tumor cells grown over the anatomical location of brain. The mouse I or II, respectively, were used for the group I or the group II aptamer injection. The lesions were outlined with black circles. **(B)** The black boxes encircled the regions of interest used to calculate the averaged radiance (p/s/cm^2/sr); the averaged radiance of the blank control served as the basal level for signal extraction. **(C)** BLI signals emitted from the tumor cells; the mouse I-IV, respectively, were used for AptB1-B4 injection. The lesions were outlined with black circles. **(D)** Detection of the Cy5-AptB1 signals in the anatomical location of brain at 0.5 hr, 1 hr, 3 hr, and 6 hr after injection, in a crescendo pattern.

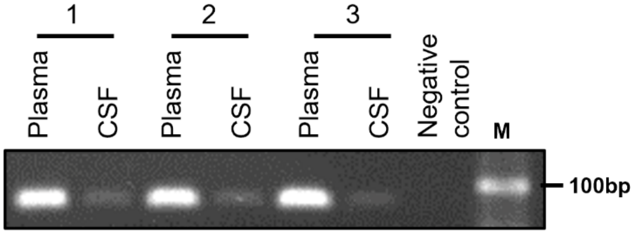

	Area (pixels)		
	CSF	Plasma	CSF/plasma Ratio (%)
1	681.11	6406.50	10.63
2	792.94	6516.08	12.17
3	657.82	7091.79	9.28
Mean (SD)	710.62 (58.98)	6671.46 (300.57)	10.69 (1.18)

Figure S2. AptBCis1 BBB penetration rate. The analysis with agarose gel electrophoresis on the PCR-amplified samples suggested a 10% CSF to plasma ratio of AptBCis1. The number of PCR cycle was 30.

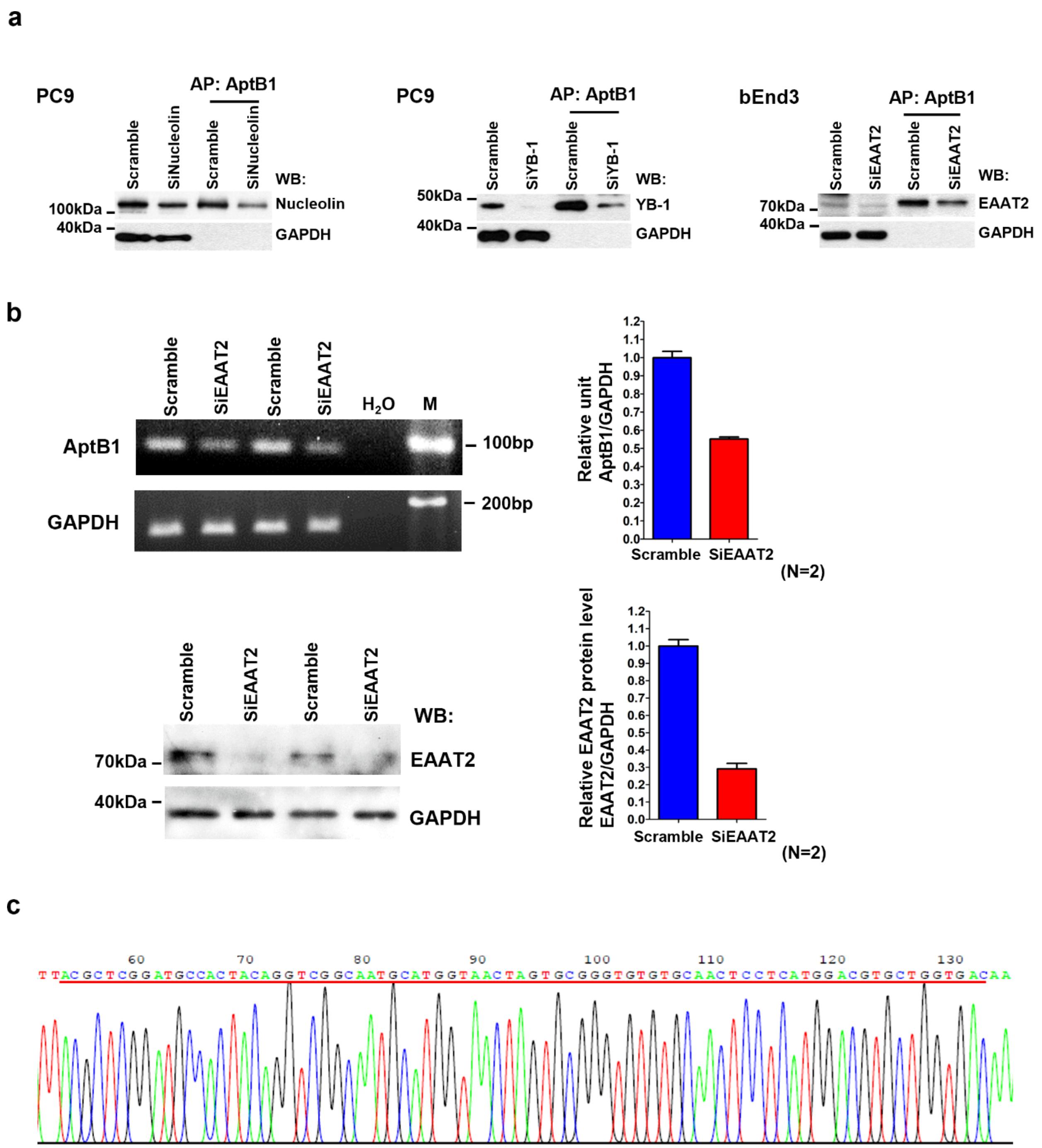

Figure S3. Results of antibody specificity, effects of EAAT2 knockdown on AptB1 uptake by bEnd3 cells, and Sanger sequencing of the AptB1 sequence. (A) The specificity of Nucleolin, YB-1 and EAAT2 antibodies was verified by immunoblots and AP-immunoblots with cell lysates prepared from cells with and without SiRNA treatment. **(B)** Knockdown of EAAT2 led to decreased cellular uptake of AptB1 by bEnd3 cells. **(C)** Sequence confirmation of the AptB1 PCR amplicons derived from the nucleus fraction by Sanger sequencing.

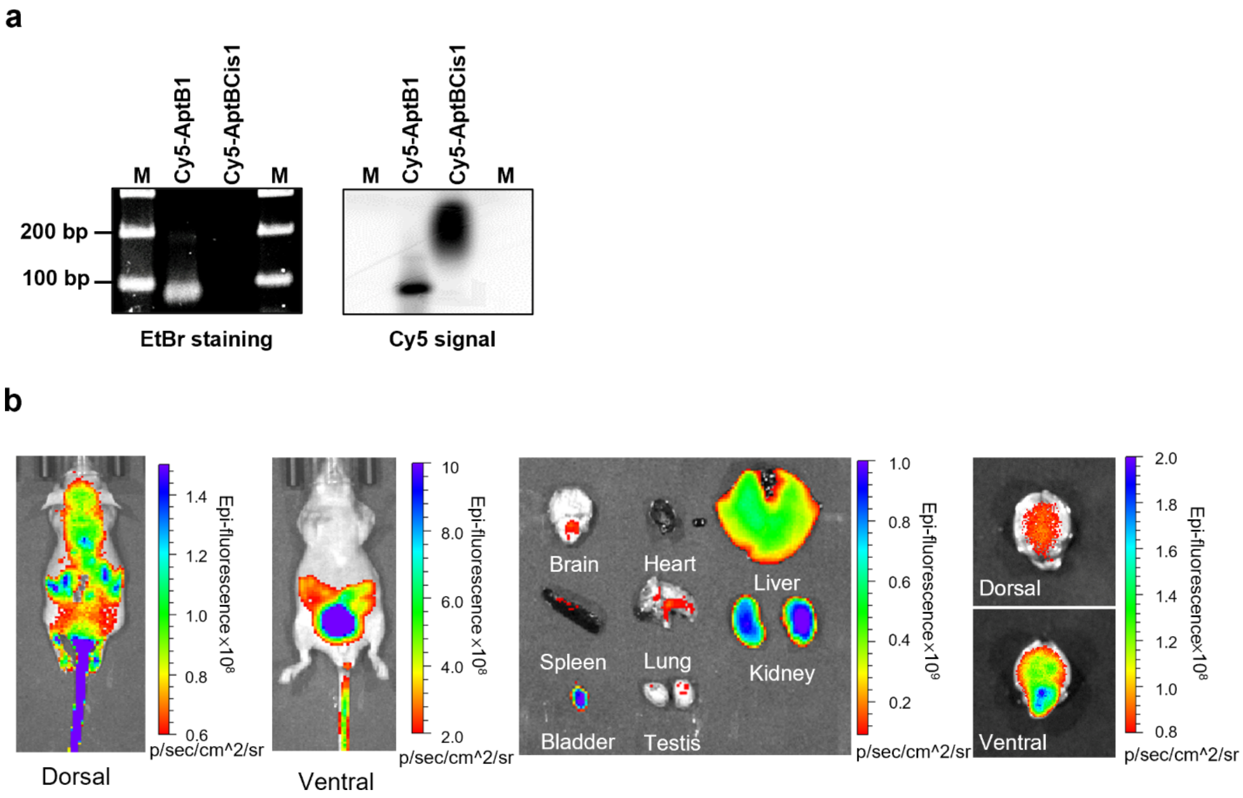

Figure S4. The biodistribution of AptBCis1 in nude mice. (A) Successful conjugation of Cy5-AptB1 with cisplatin. The Cy5 signals were obtained by Typhoon 9410 (GE). **(B)** The IVIS images taken at 30 minutes after Cy5-AptBCis1 intravenous administration illustrated signals over the bladder, kidney, liver, brain and lung in a decrescendo pattern.
